# Small-molecule active pharmaceutical ingredients of approved cancer therapeutics inhibit human aspartate/asparagine-β-hydroxylase

**DOI:** 10.1016/j.bmc.2020.115675

**Published:** 2020-10-15

**Authors:** Lennart Brewitz, Anthony Tumber, Xiaojin Zhang, Christopher J. Schofield

**Affiliations:** a Chemistry Research Laboratory, University of Oxford, 12 Mansfield Road, OX1 3TA Oxford, United Kingdom; b Laboratory of Drug Design and Discovery, Department of Chemistry, China Pharmaceutical University, Nanjing 211198, China

**Keywords:** Aspartate/asparagine-β-hydroxylase (AspH, BAH, HAAH), 2-oxoglutarate (α-ketoglutarate) dependent oxygenase, drug repositioning, bleomycin A_2_, B-cell lymphoma-2 (Bcl-2) inhibitors, (R)‑gossypol (AT-101)

## Abstract

Human aspartate/asparagine-β-hydroxylase (AspH) is a 2-oxoglutarate (2OG) dependent oxygenase that catalyses the hydroxylation of Asp/Asn-residues of epidermal growth factor-like domains (EGFDs). AspH is reported to be upregulated on the cell surface of invasive cancer cells in a manner distinguishing healthy from cancer cells. We report studies on the effect of small-molecule active pharmaceutical ingredients (APIs) of human cancer therapeutics on the catalytic activity of AspH using a high-throughput mass spectrometry (MS)-based inhibition assay. Human B-cell lymphoma-2 (Bcl-2)-protein inhibitors, including the (*R*)-enantiomer of the natural product gossypol, were observed to efficiently inhibit AspH, as does the antitumor antibiotic bleomycin A_2_. The results may help in the design of AspH inhibitors with the potential of increased selectivity compared to the previously identified Fe(II)-chelating or 2OG-competitive inhibitors. With regard to the clinical use of bleomycin A_2_ and of the Bcl-2 inhibitor venetoclax, the results suggest that possible side-effects mediated through the inhibition of AspH and other 2OG oxygenases should be considered.

## Graphical abstract

**Figure:**
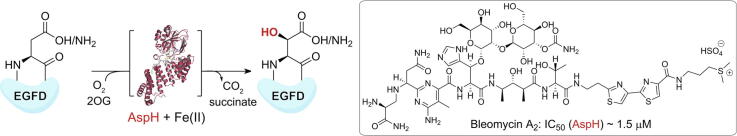

## 1 Introduction

**Fig. 1:**
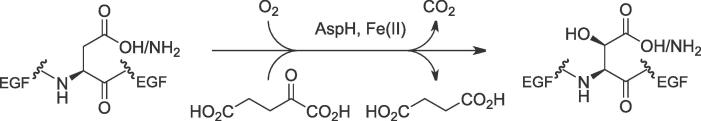
The stoichiometry of the AspH-catalysed post-translational hydroxylation of Asn- and Asp-residues in epidermal growth factor (EGF)-like domains.

Human aspartate/asparagine-β-hydroxylase (AspH, BAH, HAAH) belongs to the family of 2-oxoglutarate (2OG) dependent oxygenases^1^ and catalyses the post-translational hydroxylation of specific aspartyl- and asparaginyl-residues in human epidermal growth factor-like domains (EGFDs) using 2OG and O_2_ as co-substrates and Fe(II) as a cofactor (Fig. 1).^2^, ^3^ Hypoxia is reported to regulate the expression levels of human AspH^4^, ^5^ and upregulated levels of AspH have been detected on the cell surface of invasive human cancers such as hepatocellular carcinoma,^6^, ^7^ breast cancer,^8^ and pancreatic cancer.^9^ AspH is reported to retain its catalytic activity on the cancer cell surface.^10^ The upregulation of AspH and its translocalization from the endoplasmic reticulum (ER) membrane to the cell surface in cancer cells correlates with enhanced cell motility and metastatic spread resulting in a reduced lifespan of the affected patients.^11^, ^12^ Studies on naturally occurring mutations of human AspH presumably resulting in inactive AspH (Traboulsi syndrome)^13^, ^14^, ^15^, ^16^ and animal models^8^, ^17^, ^18^ suggest that AspH might affect cell motility through the notch signalling pathway. Despite the combined evidence suggesting that AspH is a promising medicinal chemistry target for the development of small-molecule-based cancer therapeutics, comparatively few AspH inhibition studies using small-molecules are reported, with most of these relying either on the use of likely non-selective^19^, ^20^ or partially selective^21^ small-molecules or on the use of l-ascorbic acid (LAA)-derived small-molecules.^18^, ^22^, ^23^, ^24^ This likely reflects the historic lack of simple and reliable assays to monitor recombinant human AspH activity *in vitro*. Pioneering assays employed (native) partially purified bovine or murine AspH and monitored AspH activity by analysing either 2OG turnover or the amino acid content of EGFD substrate peptides after acidic hydrolysis using mass spectrometry (MS).^20^, ^25^, ^26^, ^27^

Recently, we reported a catalytically active truncated form of recombinant human AspH, His_6_-AspH_315-758_, comprising its catalytic oxygenase domain and the adjacent tetratricopeptide repeat (TPR) domain, which was suitable to perform *in vitro* turnover assays and crystallographic experiments.^28^ We showed that AspH catalyses the Asp/Asn-hydroxylation of EGFDs with a non-canonical disulfide pattern (Cys 1–2, 3–4, 5–6; Supporting Fig. S1a) rather than with the well-characterized canonical disulfide pattern (Cys 1–3, 2–4, 5–6; Supporting Fig. S1a),^28^ which has been predominantly found in EGFD-bearing proteins.^29^ Cyclic peptides were designed to mimic the central non-canonical (Cys 3–4) EGFD disulfide macrocycle and were applied as stable AspH substrate analogues in kinetic experiments, the results of which suggest that AspH activity may be unusually susceptible to limiting oxygen availability and thus that AspH might be involved in the physiologically relevant hypoxic response.^30^ High-throughput solid phase extraction coupled to mass spectrometry (SPE-MS) AspH inhibition assays using a stable substrate analogue were developed and employed to identify potent AspH inhibitor templates.^21^

The development of novel human therapeutics is time-consuming and often compromised by the failure of target and lead candidates to be approved for therapeutic use in humans.^31^, ^32^ The repositioning of small-molecule active pharmaceutical ingredients (APIs) of approved human therapeutics, meaning the repurposing of APIs against other than the approved indications, constitutes a potential alternative as multiple phases of the *de novo* drug discovery process can be bypassed.^31^ One aspect of API repositioning involves the identification of previously unrecognized API-protein interactions.^31^ Hence, profiling of approved small-molecule APIs against human enzymes other than the reported target enzymes is desirable.^31^, ^33^ This also helps to identify inhibitor scaffolds for structure activity studies and to define undesired off-target side effects of approved APIs. We thus aimed to screen APIs against AspH, with a special interest in cancer therapeutics.

We report the effects of 316 small-molecule APIs, which are either components of approved human cancer therapeutics or of human cancer therapeutics under current or previous clinical investigation, on the catalytic activity of AspH, employing SPE-MS inhibition assays. Both natural products and clinical inhibitors of human anti-apoptotic B-cell lymphoma-2 (Bcl-2)^34^, ^35^ proteins were identified to efficiently inhibit AspH.

## 2 Results

### 2.1 Evaluation of small-molecule cancer APIs for AspH inhibition

Initially, a compound library composed of 316 small-molecules (the Approved Oncology Drugs Set of the National Cancer Institute/the National Institutes of Health Developmental Therapeutics Program combined with the TDI Expanded Oncology Drug Set of the Target Discovery Institute, University of Oxford), which are either APIs of approved human cancer therapeutics or of human cancer therapeutics under current or previous clinical investigation, was investigated for AspH inhibition under the previously established AspH inhibition assay conditions.^36^ AspH substrate- (hFX-CP_101–119_; Supporting Fig. S1b), 2OG-, and Fe(II)-concentrations close to their Michaelis (*K*_m_) constants^30^ were employed and substrate depletion/product formation (+16 Da) was monitored by SPE-MS to identify potent AspH inhibitors (Supporting Fig. S2 and Supporting Data Sheet). At a fixed inhibitor concentration (20 μM), eleven compounds inhibited >80% AspH activity with respect to DMSO and pyridine-2,4-dicarboxylic acid (2,4-PDCA; Table 1, entry 1)^19^, ^20^, ^36^ negative and positive controls, respectively (Table 1).

**Table 1:** Small-molecules of the cancer API set inhibiting >80% AspH activity.

	Inhibitor	a^,^^b^AspH inhibition at 20 μM [%]	bIC_50_ [μM]	Reported biochemical target
1	Pyridine-2,4-dicarboxylic acid (2,4-PDCA)	100.0 ± 0.6^c^	0.02 ± 0.01^d^	–
2	(*R*)-Gossypol (AT-101)	101.2 ± 2.3	0.25 ± 0.01	Broad-spectrum inhibitor of anti-apoptotic Bcl-2 proteins^37^, ^38^, ^39^
3	Bleomycin A_2_^e^^,^^f^	97.5 ± 1.3	1.47 ± 0.42^g^	Antitumor antibiotic promoting DNA-degradation^40^, ^41^, ^42^
4	Venetoclax (ABT-199)^f^	97.2 ± 0.2	1.40 ± 0.14^g^	Selective inhibitor of the anti-apoptotic protein Bcl-2^43^, ^44^
5	Belinostat (PXD101)^f^	97.1 ± 0.2	7.49 ± 1.26^g^	Broad-spectrum HDAC inhibitor^45^
6	Midostaurin (PKC412)^f^	91.7 ± 0.9	9.43 ± 3.07	Inhibitor of activating mutations of the FMS-like tyrosine kinase-3 (FLT3)^46^, ^47^
7	Tubacin	90.3 ± 3.5	4.12 ± 0.50	Selective inhibitor of HDAC6^48^
8	Navitoclax (ABT-263)	90.1 ± 0.1	1.03 ± 0.12	Inhibitor of anti-apoptotic proteins Bcl-2, Bcl-X_L_, and Bcl-w^49^
9	Mithramycin A^h^	86.9 ± 2.1	9.50 ± 0.28^g^	Antitumor antibiotic inhibiting gene transcription^50^
10	Plicamycin^h^	85.4 ± 3.9	–	–
11	Obatoclax (GX15-070)^f^^,^^i^	82.0 ± 0.6	13.2 ± 3.1	Broad-spectrum inhibitor of anti-apoptotic Bcl-2 proteins^51^, ^52^
12	Vemurafenib (PLX4032)^f^	81.1 ± 0.5	12.9 ± 1.6^g^	Selective inhibitor of mutant (V600E) BRAF kinase^53^, ^54^
13	ABT-737	–	3.38 ± 0.34	Inhibitor of anti-apoptotic proteins Bcl-2, Bcl-X_L_, and Bcl-w^55^

a The complete screening results are shown in the Supporting Data Sheet. AspH inhibition assays (20 μM fixed inhibitor concentration) were of good quality which high signal-to-noise (S/N) and high Z'-factors^56^ (>0.8 for each plate) manifest (Supporting Fig. S2).

b Mean of two independent runs (n = 2; mean ± standard deviation, SD) using 50 nM His_6_-AspH_315–758_, 1 μM hFX-CP_101–119_ (Supporting Fig. S1b), 100 μM l-ascorbic acid (LAA), 3 μM 2OG, and 2 μM ammonium iron(II) sulfate hexahydrate (FAS, (NH_4_)_2_Fe(SO_4_)_2_·6H_2_O) in 50 mM HEPES buffer (pH 7.5, 20 °C).

c Used as a positive inhibition control (n = 64; mean ± SD).

d n = 4; mean ± SD.

e Used as the hydrogensulfate salt.

f Approved API for clinical use as a human therapeutic.

g Hill coefficient^57^ < −2.0.

h Mithramycin A and plicamycin are identical.

i Used as the methanesulfonate salt.

Strikingly, four of the most active eleven identified AspH inhibitors of the cancer API compound library are reported inhibitors of human anti-apoptotic B-cell lymphoma-2 (Bcl-2)^34^, ^35^ proteins (including Bcl-2, Bcl-X_L_, and Bcl-w): the (*R*)-enantiomer of the natural product gossypol (AT-101; Table 1, entry 2),^37^, ^38^, ^39^ Abbott-developed venetoclax^43^, ^44^ (ABT-199; Table 1, entry 4) and navitoclax^49^ (ABT-263; Table 1, entry 8), and the natural-product derived obatoclax^51^, ^52^ (GX15-070; Table 1, entry 11). Complete suppression of AspH activity was observed in case of (*R*)-gossypol, while obatoclax was the least efficient AspH inhibitor out of these four compounds (~20% AspH activity remaining at 20 μM; Table 1, entry 11). Venetoclax, which is reported to be a selective inhibitor of Bcl-2 and which is clinically approved for treating chronic lymphocytic leukaemia or small lymphocytic lymphoma,^58^ seemed to be slightly more potent in inhibiting AspH than the structurally-related navitoclax, which also inhibits other Bcl-2 family proteins (~3% versus ~10% AspH activity remaining at 20 μM).

Two out of the remaining seven identified AspH inhibitors were histone deacetylase (HDAC) inhibitors, both bearing a hydroxamate functional group, which inhibited > 90% AspH activity at 20 μM: the HDAC6-selective inhibitor tubacin^48^ (Table 1, entry 7) and the broad-spectrum HDAC inhibitor belinostat (PXD101)^45^ (Table 1, entry 5), which is an approved API for the treatment of relapsed or refractory peripheral *T*-cell lymphoma.^59^

The two small-molecule APIs midostaurin^46^, ^47^ (PKC412; Table 1, entry 6) and vemurafenib^53^, ^54^ (PLX4032; Table 1, entry 12) are kinase inhibitors. Midostaurin is reported to inhibit activating mutant forms of the human FMS-like tyrosine kinase-3 (FLT3) and is approved for the treatment of acute myeloid leukaemia bearing FLT3 activating mutations; it inhibits >90% AspH activity at 20 μM (Table 1, entry 6). Vemurafenib is reported to inhibit selectively the human serine/threonine kinase BRAF bearing an activating V600E mutation over the wild-type enzyme. It is approved for the treatment of metastatic melanoma and seems to impose a modest inhibitory effect on AspH activity (~20% AspH activity remaining at 20 μM; Table 1, entry 12).

Antitumor antibiotics are the final compound category identified to inhibit AspH, namely the clinically used compound bleomycin A_2_,^40^, ^41^, ^42^ which inhibits > 97% AspH activity at 20 μM (Table 1, entry 3), and the glyco-antibiotic mithramycin A (plicamycin; Table 1, entries 9 and 10).^50^ Mitramycin A (plicamycin) is reported to inhibit gene transcription through binding GC-rich DNA sequences and decreases the catalytic activity of AspH by ~85% at 20 μM (Table 1, entries 9 and 10).^50^ The similar potencies obtained for mitramycin A and plicamycin, which are identical compounds, reflect the excellent quality of the SPE-MS AspH inhibition assay (Supporting Fig. S2).

### 2.2 Determination of AspH inhibitory concentrations

**Fig. 2:**
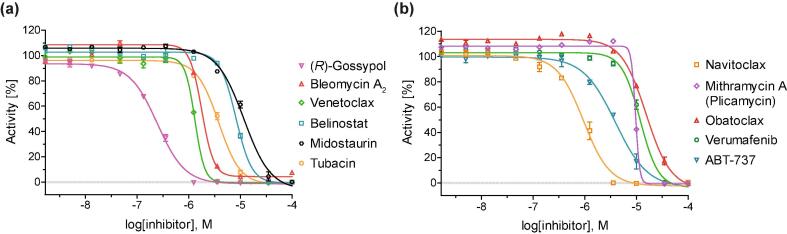
Representative dose-response curves used to determine IC_50_-values for small-molecule AspH inhibitors displaying >80% potency in the initial AspH inhibition screen. Two dose-response curves each composed of technical duplicates were independently determined using SPE-MS AspH inhibition assays. Assay conditions: 50 nM His_6_-AspH_315–758_, 1 μM hFX-CP_101–119_ (Supporting Fig. S1b), 100 μM l-ascorbic acid (LAA), 3 μM 2OG, and 2 μM Fe(II) in 50 mM HEPES buffer (pH 7.5, 20 °C). Data are shown as the mean average of two technical duplicates (n = 2; mean ± SD).

Fresh DMSO solutions of the ten identified AspH inhibitors were prepared from separately obtained commercial solids and used to determine half-maximum inhibitory concentrations (IC_50_) using substrate (hFX-CP_101-119_; Supporting Fig. S1b), 2OG-, and Fe(II)-concentrations close to their Michaelis (*K*_m_) constants (Table 1, Fig. 2).^30^ The Abbott-developed Bcl-2 inhibitor ABT-737^55^ (Table 1, entry 13) was added to the panel due to its structural similarity with navitoclax and venetoclax. The AspH inhibition assays were of good quality which high signal-to-noise (S/N) ratios and high Z'-factors^56^ (>0.75 for each plate) manifest (Supporting Fig. S3). This is in agreement with our previous AspH inhibition assays and highlights the power of the SPE-MS high-throughput screening platform to perform AspH inhibition studies.^36^

The IC_50_-values are in good agreement with the initial screening data at a fixed inhibitor concentration: (*R*)-Gossypol, bleomycin A_2_, and venetoclax all inhibited AspH with IC_50_-values of ~1 μM or below (Table 1). With the exception of navitoclax (IC_50_ ~ 1.0 μM; Table 1, entry 7), which inhibits AspH with a similar potency as venetoclax, all the other identified APIs displayed only modest potencies (~4.1 to ~13.2 μM; Table 1). ABT-737 was about three fold less potent than its derivatives navitoclax and venetoclax (IC_50_ ~ 3.4 μM; Table 1, entry 13). (*R*)-Gossypol inhibits AspH with similar potency as its Bcl-2 validated targets (*i.e.* IC_50_ (Bcl-X_L_) ~ 0.5 μM)^37^ while the Abbott-developed APIs are significantly more potent against their original Bcl-2 protein targets, even in cell-based experiments (IC_50_ < 1 nM).^34^

### 2.3 Mechanistic studies

To investigate whether the mechanisms by which the identified eleven small-molecule APIs inhibit AspH involve competition with 2OG or substrate for AspH binding or non-specific Fe(II)-chelation, their IC_50_-values were determined at elevated 2OG (~330*·K*_m_; *K*_m_ = 0.6 μM^30^), elevated substrate peptide (hFX-CP_101-119_; ~8*·K*_m_; *K*_m_ = 1.2 μM^30^) or elevated Fe(II) (~14*·K*_m_; *K*_m_ = 1.4 μM^30^) assay concentrations, respectively (Table 2). We initially studied the potent but non-selective AspH inhibitor 2,4-PDCA (Table 1, entry 1) as a positive control.^19^, ^20^, ^36^ These analyses demonstrated that 2,4-PDCA competes with 2OG for binding to the AspH active site: at an elevated 2OG concentration, 2,4-PDCA is about five times less potent than at the AspH *K*_m_-concentration for 2OG, while varying the AspH substrate peptide or Fe(II) concentrations did not affect its IC_50_-value (Table 2; entry 1). This observation is in agreement with prior crystallographic studies and biophysical experiments,^36^ indicating that this SPE-MS AspH inhibition assay set-up is useful to probe the mechanism by which the small-molecule APIs inhibit AspH.

**Table 2:** AspH inhibition by selected APIs of cancer therapeutics at high 2OG, Fe(II), or substrate peptide (hFX-CP_101–119_) concentrations.

	Inhibitor	IC_50_ [μM]^a,b^	IC_50_ [μM]^a,c^ at 200 μM 2OG	IC_50_ [μM]^a,d^ at 20 μM Fe(II)	IC_50_ [μM]^a,e^ at 10 μM substrate
1	2,4-PDCA^19^, ^20^, ^36^	0.02 ± 0.01^f^	0.10 ± 0.03^f^	0.03 ± 0.01^f^	0.02 ± 0.01
2	(*R*)-Gossypol (AT-101)^37^, ^38^, ^39^	0.25 ± 0.01	0.25 ± 0.01	0.26 ± 0.03	0.33 ± 0.02
3	^g,h^Bleomycin A_2_^40^, ^41^, ^42^	1.47 ± 0.42^i^	3.81 ± 1.48	1.99 ± 0.70	1.65 ± 0.25^i^
4	^h^Venetoclax (ABT-199)^43^, ^44^	1.40 ± 0.14^i^	1.52 ± 0.24^i^	1.29 ± 0.24^i^	1.57 ± 0.21^i^
5	^h^Belinostat (PXD101)^45^	7.49 ± 1.26^i^	11.2 ± 4.0^i^	3.10 ± 1.44^i^	4.77 ± 0.10^i^
6	^h^Midostaurin (PKC412)^46^, ^47^	9.43 ± 3.07	12.4 ± 2.9	7.28 ± 0.90	6.02 ± 1.32
7	Tubacin^48^	4.12 ± 0.50	5.69 ± 0.70	4.91 ± 1.63	3.57 ± 0.10
8	Navitoclax (ABT-263)^49^	1.03 ± 0.12	1.33 ± 0.17	0.80 ± 0.42	1.21 ± 0.04
9	Mithramycin A (plicamycin)^50^	9.50 ± 0.28^i^	12.2 ± 1.4^i^	Inactive	3.39 ± 0.99^i^
10	^h,j^Obatoclax (GX15-070)^51^, ^52^	13.2 ± 3.1	16.4 ± 4.6	11.9 ± 0.8^i)^	9.44 ± 3.16^i)^
11	^h^Vemurafenib (PLX4032)^53^, ^54^	12.9 ± 1.6^i^	15.1 ± 0.2^i^	10.5 ± 1.9^i^	16.9 ± 4.4^i^
12	ABT-737^55^	3.38 ± 0.34	4.47 ± 1.32	2.92 ± 0.88	2.99 ± 0.35

a) Mean of two independent runs (n = 2; mean ± SD); b) Assay conditions: 50 nM His_6_-AspH_315–758_, 1 μM hFX-CP_101–119_ (Supporting Fig. S1b), 100 μM LAA, 3 μM 2OG, and 2 μM FAS, in 50 mM HEPES buffer (pH 7.5, 20 °C); c) using 200 μM 2OG instead of 3 μM 2OG; d) using 20 μM Fe(II) instead of 2 μM Fe(II); e) using 10 μM hFX-CP_101–119_ and 10 μM 2OG instead of 1 μM hFX-CP_101–119_ and 3 μM 2OG; f) n = 4; mean ± SD; g) used as the hydrogensulfate salt; h) approved API for clinical use as a human therapeutic; i) Hill coefficient^57^ < −2.0; j) used as the methanesulfonate salt. The AspH inhibition assays were of good quality which high S/N ratios and Z'-factors^56^ (>0.75 for each plate) indicate (Supporting Fig. S3).

By contrast with the results for 2,4-PDCA, the IC_50_-values of the small-molecule APIs do not differ notably at elevated 2OG, substrate peptide or Fe(II) concentrations, within the experimental error (Table 2). These results suggest that the APIs do not seem to inhibit AspH in a 2OG or substrate competitive manner or through lowering the available Fe(II) concentration. However, this analysis is not conclusive, as for some of the AspH inhibition curves, *i.e.* with bleomycin A_2_, venetoclax, belinostat, mithramycin A (plicamycin), obatoclax, and verumafenib, comparably low Hill coefficients^57^ (<-2.0) were observed, with mithramycin displaying the most pronounced effect (Fig. 2). Low Hill coefficients, which diverge notably from the ‘ideal’ value (~−1), can be observed if enzymes contain multiple inhibitor binding sites.^60^, ^61^ Low Hill coefficients can also be a result of inhibitor aggregation,^62^, ^63^ which is a well-established phenomenon in inhibitor identification from high-throughput compound library screenings;^63^, ^64^, ^65^ the small-molecules can aggregate to form colloidal particles^66^ which then can associate with the enzyme and trigger denaturation.^67^

Vemurafenib is reported to form colloidal particles in aqueous solutions and in cell-cultures,^68^, ^69^ indicating that aggregation might account for the observed inhibitory effect of some of the identified small-molecule AspH inhibitors. The AspH inhibition assays were thus performed in the presence of detergents^70^ with the aim of suppressing small-molecule aggregation. However, both the tested zwitterionic detergent CHAPS^71^ and the non-ionic detergent triton-X100^72^, ^73^ suppressed AspH substrate peptide ionization at concentrations typically used to suppress aggregation; the use of detergents was hence not compatible with the SPE-MS AspH inhibition assay. An orthogonal binding assay was therefore applied to elucidate if the identified AspH inhibitors bind AspH. AspH melting temperatures (T_m_) were assayed in the presence of the small-molecules using differential scanning fluorimetry (Supporting Fig. S4). Several identified AspH inhibitors were incompatible with the assay conditions due to intrinsic compound fluorescence interfering with the excitation/emission wavelengths of SYPRO orange; all the other inhibitors did not show a substantial effect on the AspH T_m_ when compared to the reported 2OG competitor 2,4-PDCA, which caused stabilization of AspH (ΔT_m_ = 3.2 ± 0.4 °C; Supporting Fig. S4).

In the case of mithramycin A (plicamycin), Fe(II)-chelation could account for some of its observed inhibitory effect, because no inhibition (*i.e.* IC_50_ > 50 μM) of AspH was observed at higher Fe(II) assay concentrations (Table 2, entry 9). This proposal is consistent with the chemical structure of mithramycin A, which bears functional groups that can engage in metal chelation (Table 1, entry 9), with its mode of action that requires divalent metal cations to form inhibitor-metal cation-DNA ternary complexes,^50^ and with its negligible effect on the AspH T_m_ (ΔT_m_ = 0.4 ± 0.2 °C; Supporting Fig. S4).

The potency of bleomycin A_2_ did not depend on the Fe(II) assay concentration (Table 2, entry 3), an observation which is notable given that bleomycin A_2_ is reported to require activation by Fe(II) and O_2_ in order to exert its medicinal function as DNA-cleavage agent.^42^, ^74^^-^^75^ Hill coefficient analysis for bleomycin A_2_ AspH inhibition curves indicates that compound aggregation alone is unlikely to account for the observed AspH inhibition; to our knowledge, bleomycin A_2_ has not been reported to form colloidal aggregates in aqueous solutions. One possible explanation for its inhibitory effect could be that activated bleomycin A_2_, which is a known oxidant,^42^ alters the assay redox equilibrium in the assay mixture, and thus indirectly inhibits AspH, which is known to be sensitive towards subtle changes in the redox environment.^30^ Although further work is required, this possibility is in agreement with the results that altered 2OG, substrate peptide or Fe(II) assay concentrations did not affect bleomycin A_2_ potency (Table 2, entry 3) and that bleomycin A_2_ did not seem to bind AspH efficiently (ΔT_m_ = −0.8 ± 0.4 °C; Supporting Fig. S4).

The efficiencies of the four most potent small-molecule AspH inhibitors identified apart from bleomycin A_2_ (*i.e.* (*R*)-gossypol, venetoclax, navitoclax, and ABT-737), which are all reported inhibitors of Bcl-2 proteins, did not seem to depend on 2OG, substrate peptide or Fe(II) assay concentrations (Table 2). Even though the structures of these AspH inhibitor resemble those of reported small-molecule aggregators,^76^, ^77^ the Hill coefficient analysis for their AspH inhibition curves (Fig. 2) together with their slight effect on the AspH T_m_ (Supporting Fig. S4) indicates that mechanisms other than aggregation might account for their observed inhibition of AspH. As co-crystallization experiments with AspH with the Bcl2-inhibitors have been unsuccessful, molecular docking studies were performed to predict the binding of the four reported Bcl-2 inhibitors to AspH. The results indicate that binding of the four small-molecules to defined AspH residues is feasible, at least in principle (Supporting Figs. S5–S8).

## 3 Discussion

Small-molecule AspH probes for use *in vivo* should possess drug-like properties^78^ to facilitate cell-based biochemical as well as animal model studies. A straight-forward way of identifying drug-like AspH probes is to investigate small-molecule APIs of approved human therapeutics for AspH inhibition, as drug repositioning constitutes a successful general strategy to develop novel enzyme inhibitors.^31^, ^33^ Therefore, we investigated the inhibitory effect of 316 small-molecules, which are either APIs of approved human cancer therapeutics or of human cancer therapeutics under current or previous clinical investigation, against AspH using our previously reported high-throughput SPE-MS AspH inhibition assay (Supporting Fig. S2 and Supporting Data Sheet).^36^ The focus was on APIs of cancer therapeutics as the identification of potential side-effects of these APIs through inhibiting AspH could render alternative explanations of their biological effects.

The high-throughput screen revealed that eleven small-molecule APIs, categorised into reported inhibitors of Bcl-2 family proteins, hydroxamate-containing HDAC inhibitors, kinase inhibitors, and antitumor antibiotics, effected AspH activity (Table 1). Hydroxamates, including HDAC inhibitors, have been reported to inhibit 2OG oxygenases.^79^, ^80^, ^81^ However, efficient AspH inhibition was only observed for the antitumor antibiotic bleomycin A_2_^40^, ^41^, ^42^ and for four Bcl-2 inhibitors, *i.e.* (*R*)-gossypol,^37^, ^38^, ^39^ venetoclax,^43^, ^44^ navitoclax,^49^ and ABT-737,^55^ while the remaining identified AspH inhibitors displayed only moderate potency (Table 1). These five small-molecules efficiently inhibited AspH at different 2OG assay concentrations (3 μM and 200 μM 2OG; Table 2) which are in the range of reported physiological 2OG levels in human plasma (9–12 μM 2OG)^82^ and cells (>1mM 2OG).^83^, ^84^, ^85^

Although bleomycin A_2_ efficiently inhibits AspH (IC_50_ ~ 1.5 μM; Table 2, entry 3), its precise mechanism of inhibition requires further work. Some evidence suggests that bleomycin A_2_ aggregates in solution (Fig. 2), which might cause AspH inhibition. Alternatively, reactive oxygen species (ROS) generated by bleomycin A_2_ might account for its observed inhibitory effect. AspH activity has been previously shown to decrease in the presence of ROS^30^ and bleomycin A_2_ is reported to form ROS in the presence of Fe(II) and O_2_.^42^, ^74^^-^^75^ This proposal for the mode of action of bleomycin A_2_ in inhibiting AspH is consistent with the result that bleomycin A_2_ does not seem to bind AspH efficiently (ΔT_m_ ~ −0.8 °C; Supporting Fig. S4). This proposal would potentially complement previously determined mechanisms how natural products inhibit AspH, *i.e.* 2OG-competition for binding the AspH active site (*e.g. N*-oxalylglycine,^28^, ^36^, ^86^ pyridine-2,3-dicarboxylic acid21., 87) and Fe(II)-chelation (*e.g.* L-mimosine^36^, ^88^). Studies on the inhibition of the activities of other 2OG oxygenases by bleomycin A_2_, both in cells and with isolated enzymes, are thus of interest. Given the role of bleomycin A_2_ in tumour treatment, investigating its effect on 2OG oxygenases involved in DNA damage repair and DNA/RNA modification,^89^, ^90^ including human homologues of alkylated DNA repair protein (AlkB),^91^, ^92^, ^93^ is of particular interest.

Out of the four Bcl-2 inhibitors found to inhibit AspH, the (*R*)-enantiomer of the natural product gossypol (AT-101; Table 1, entry 2),^37^, ^38^, ^39^ which was originally obtained from cottonseed extracts and which has been investigated as a male contraceptive agent,^94^, ^95^ inhibits AspH with similar efficiency (IC_50_ (AspH) ~ 0.3 μM; Table 2, entry 2) as its validated Bcl-2 target enzymes, *i.e.* Bcl-2, Bcl-X_L_, and Bcl-w (IC_50_ (Bcl-X_L_) ~ 0.5 μM).^37^ However, care should be taken when comparing the absolute IC_50_-values as these were obtained using different assay techniques, *i.e.* SPE-MS for AspH (50 nM enzyme concentration) and fluorescence polarisation for Bcl-X_L_ (30 nM enzyme concentration).^37^ Although molecular docking studies suggest that this natural product may inhibit AspH by directly binding to the enzyme (Supporting Figs. S5 and S8), it has been reported that gossypol induces the formation of reactive oxygen species, which, as for bleomycin A_2_, might also account for some of its observed bioactivity against AspH.^96^ The clinical use of gossypol is associated with numerous side-effects, which may have hampered its progression from therapeutic studies into clinical use as an anticancer agent and suggest that this natural product is a non-specific inhibitor acting against different enzyme-classes apart from Bcl-2 proteins.^97^ AspH selectivity counter-screens could thus be performed in order to develop gossypol-derived small-molecule Bcl-2 inhibitors, such as TW-37^98^ and sabutoclax^99^, ^100^, into safer medicines.

The Abbott-developed small-molecule Bcl-2 inhibitors venetoclax^43^, ^44^ (IC_50_ ~ 1.4 μM; Table 2, entry 4) and navitoclax^49^ (IC_50_ ~ 1.0 μM; Table 2, entry 8) were efficient AspH inhibitors, while ABT-737^55^ was slightly less potent (IC_50_ ~ 3.4 μM; Table 2, entry 12). Biophysical binding assays and molecular docking studies indicate that the mechanism by which the three small-molecule Bcl-2 inhibitors impede AspH activity may involve binding to the enzyme (Supporting Figs. S4 and S6–S8), which could for example occur in an allosteric manner. Optimizing the interactions of the Abbott-developed small-molecules with AspH while minimizing their reported interactions with the Bcl-2 family proteins should foster the design of AspH-selective inhibitors as part of a detailed structure–activity relationship study.

The results are also of relevance to the clinical use of venetoclax,^43^, ^44^ which is an approved API for the treatment of chronic lymphocytic leukaemia and small lymphocytic lymphoma.^58^ AspH inhibition could to some extent account for the observed anti-cancer effects of venetoclax, even though this API inhibits its validated target enzyme, Bcl-2, significantly more efficient than AspH (~1000 fold selectivity).^34^ Hill coefficient^57^ analysis of AspH inhibition curves suggests that venetoclax might aggregate in solution and form colloidal particles, which could account for its observed inhibitory effect (Table 2).^62^ This raises the question if, and to what extent, this small-molecule API approved for clinical applications may form aggregates in buffered aqueous solutions or human body fluids. It has been shown that other APIs of approved therapeutics form colloidal aggregates in a fluid-dependent manner, which can even be stable in serum over a prolonged period of time.^68^, ^101^ Aggregate formation can result in reduced cellular API uptake, thus compromising inhibitor efficiency.^68^, ^102^, ^103^, ^104^, ^105^ AspH levels are upregulated on the cell surface of cancer cells,^10^, ^11^, ^12^ while, in order to induce cellular apoptosis through the mitochondrial pathway by inhibiting Bcl-2, venetoclax has to penetrate the cell wall.^34^, ^35^ An accumulation of venetoclax outside the cell might compensate for its comparably lower efficiency to inhibit AspH.

## 4 Conclusions

The present study validates the robustness of SPE-MS AspH inhibition assays in combination with biophysical techniques to identify drug-like small-molecules as AspH probes and novel structures for inhibitor development. Natural products and approved APIs for human therapy have been identified that inhibit AspH. Although further work is required to investigate their mode of action, the observation of AspH inhibition by bleomycin A_2_, venetoclax, and (*R*)-gossypol (AT-101) could be of utility in the design of selective small-molecule AspH inhibitors for use in investigating its biological roles and its utility as a medicinal chemistry target. Studies on the role of bleomycin A_2_ and related drugs on 2OG oxygenases involved in DNA damage repair are of interest.

## 5 Materials and methods

### 5.1 General information

Milli-Q ultrapure (MQ-grade) water was used for buffers; LCMS grade solvents (Merck) were used for solid phase extraction (SPE)-MS. Access to a compound library composed of 316 small-molecules (the Approved Oncology Drugs Set of the National Cancer Institute/the National Institutes of Health Developmental Therapeutics Program combined with the TDI Expanded Oncology Drug Set of the Target Discovery Institute of the University of Oxford), in form of inhibitor solutions in DMSO, was provided by the Target Discovery Institute, Oxford (the individual compounds are listed in the Supporting Data Sheet). The most potent AspH inhibitors identified in the initial library screen were separately obtained from commercial sources (Sigma-Aldrich, Tocris) to perform dose–response experiments.

### 5.2 Recombinant AspH production and purification

*N*-Terminally His_6_-tagged human AspH_315–758_ (His_6_-AspH_315–758_) was produced in *Escherichia coli* BL21 (DE3) cells using a pET-28a(+) vector and purified by Ni(II)-affinity chromatography (HisTrap HP column, GE Healthcare; 1 mL/min flow rate) and size-exclusion chromatography (HiLoad 26/60 Superdex 75 pg 300 mL column; 1 mL/min) using an ÄKTA Pure machine (GE Healthcare), as previously reported.^28^, ^30^ AspH was > 95% pure by SDS-PAGE and MS analysis and had the anticipated mass (*m*/*z* calculated for His_6_-AspH_315–758_: 54519 Da, found: 54519 Da). Purified AspH was stored in 50 mM HEPES buffer (pH 7.5, 150 mM NaCl) at a concentration of 125 μM at −78 °C; fresh aliquots were used for all AspH inhibition assays.

### 5.3 AspH substrate synthesis

The synthetic thioether linked cyclic peptide hFX-CP_101-119_^28^ (Supporting Fig. S1b) was used as an AspH substrate; it was designed based on 19 EGFD1 amino acid residues of the sequence of human coagulation factor X (hFX amino acids 101–119), which is a reported cellular AspH substrate.^106^ hFX-CP_101-119_ was synthesized as reported by an intramolecular thioetherification cyclization reaction from the corresponding linear peptide (d-Ala replacing Cys101_hFX_ and Ser replacing Cys112_hFX_);^30^ it was prepared with a C-terminal amide.

### 5.4 AspH inhibition assays

AspH inhibition assays were performed at 2OG, Fe(II), and hFX-CP_101-119_ concentrations close to the relevant *K*_m_-values as described.^36^ For the most active AspH inhibitors identified, inhibition assays were also performed at elevated 2OG (330*·K*_m_; *K*_m_ = 0.6 μM^30^), substrate peptide (hFX-CP_101–119_; 8*·K*_m_; *K*_m_ = 1.2 μM^30^) or Fe(II) (14*·K*_m_; *K*_m_ = 1.4 μM^30^) concentrations, respectively. Co-substrate/cofactor stock solutions (l-ascorbic acid, LAA: 50 mM in MQ-grade water; 2OG: 10 or 100 mM in MQ-grade water; ammonium iron(II) sulfate hexahydrate, FAS, (NH_4_)_2_Fe(SO_4_)_2_·6H_2_O: 400 mM in 20 mM HCl diluted to 1 or 10 mM in MQ-grade water) were freshly prepared from commercial solids (Sigma Aldrich) on the day the assays were performed.

Solutions of the bioactive small-molecules (100% DMSO) were dry dispensed across 384-well polypropylene assay plates (Greiner) using an ECHO 550 acoustic dispenser (Labcyte). DMSO and 2,4-PDCA^19^, ^20^, ^36^ were used as negative and positive inhibition controls, respectively. The aqueous DMSO concentration was kept constant at 0.5%_v/v_ throughout all experiments (using the DMSO backfill option of the acoustic dispenser). Initial screening of the cancer API compound library was performed at a fixed compound concentration (20 μM). For dose–response experiments, the AspH inhibitors were dry dispensed in a 3-fold and 11-point dilution series using an acoustic dispenser (100 μM top concentration). 2,4-PDCA was used as a positive control;^36^ its IC_50_-value was determined on each assay plate to confirm the assay quality. Each reaction was performed in technical duplicates in adjacent wells on the assay plates; additionally, assays were performed in two independent duplicates on different days using fresh reagents.

An Enzyme Mixture (25 μL/well), containing 0.1 μM His_6_-AspH_315–758_ in 50 mM HEPES buffer (pH 7.5), was dispensed across the inhibitor-containing 384-well assay plates with a multidrop dispenser (ThermoFischer Scientific) at 20 °C under ambient atmosphere. The plates were subsequently centrifuged (1000 rpm, 30 s) and incubated for 15 min at 20 °C. A Substrate Mixture (25 μL/well), containing 2.0 μM hFX-CP_101–119_, 200 μM LAA, 6.0 μM 2OG, and 4.0 μM FAS in 50 mM HEPES buffer (pH 7.5), was added using the multidrop dispenser. Note: The multidrop dispenser ensured proper mixing of the Enzyme and the Substrate Mixtures which was essential for assay reproducibility. The plates were centrifuged (1000 rpm, 30 s) and after incubating for 7 min, the enzyme reaction was stopped by the addition of 10%_v/v_ aqueous formic acid (5 μL/well). The plates were centrifuged (1000 rpm, 60 s) and analysed by MS.

AspH inhibition assays at elevated 2OG (330*·K*_m_: 400 μM 2OG in the Substrate Mixture), substrate peptide (8*·K*_m_: 20 μM hFX-CP_101–119_ and 20 μM 2OG in the Substrate Mixture) or Fe(II) (14*·K*_m_: 40 μM FAS in the Substrate Mixture) assay concentrations were performed in a similar manner. However, the enzyme reaction was stopped after incubating for 6 rather than 7 min when the reaction was performed at an elevated 2OG or Fe(II) concentration, and after incubating for 35 min when the reaction was performed at an elevated AspH substrate peptide concentration.

MS-analyses were performed using a RapidFire RF 365 high-throughput sampling robot (Agilent) attached to an iFunnel Agilent 6550 accurate mass quadrupole time-of-flight (Q-TOF) mass spectrometer operated in the positive ionization mode. Assay samples were aspirated under vacuum for 0.4 s and loaded onto a C4 SPE cartridge. After loading, the C4 SPE cartridge was washed with 0.1%_v/v_ aqueous formic acid to remove non-volatile buffer salts (5 s, 1.5 mL/min). The peptide was eluted from the SPE cartridge with 0.1%_v/v_ aqueous formic acid in 85/15_v/v_ acetonitrile/water into the mass spectrometer (5 s, 1.25 mL/min) and the SPE cartridge re-equilibrated with 0.1%_v/v_ aqueous formic acid (1 s, 1.25 mL/min). The mass spectrometer parameters were: capillary voltage (4000 V), nozzle voltage (1000 V), fragmentor voltage (365 V), gas temperature (280 °C), gas flow (13 L/min), sheath gas temperature (350 °C), sheath gas flow (12 L/min). The *m*/*z* + 2 charge states of the cyclic peptide (substrate) and the hydroxylated cyclic peptide (product) were used to extract ion chromatogram data, peak areas were integrated using RapidFire Integrator software (Agilent). The data were exported into Microsoft Excel and used to calculate the % conversion of the hydroxylation reaction using the equation: % conversion = 100 × (integral product cyclic peptide)/(integral substrate cyclic peptide + integral product cyclic peptide). Normalized dose–response curves (2,4-PDCA and DMSO controls) were obtained from the raw data by non-linear regression (GraphPad Prism 5) and used to determine IC_50_-values. The standard deviation (SD) of two independent IC_50_ determinations (n = 2) was calculated using GraphPad Prism 5. Z’-factors and S/N ratios were calculated according to the cited literature using Microsoft Excel (Supporting Fig. S3).^56^

### 5.5 AspH differential scanning fluorimetry (DSF) assays

A mixture of SYPRO orange (1‰_v/v_) and His_6_-AspH_315–758_ (4 μM) in 50 mM aqueous HEPES buffer (pH 7.5, 150 mM NaCl, 50 μM NiCl_2_) was carefully pipetted into a 96-well VWR PCR-plate (38 μL per well). An inhibitor solution (0.8 mM in DMSO) or a negative control sample (pure DMSO) was added to each well (2 μL per well, final inhibitor concentration of 40 μM) and the resulting solutions were gently mixed using a pipette. The plate was sealed with optical tape (Bio-Rag, iCycler iQ), centrifuged (5 *sec*, 1000 rpm) and placed into a C1000Touch Thermal Cycler equipped with a CFX96 Real-Time System (Bio-Rad). The instrument was heated with a rate of 1 °C per cycle (starting at an initial temperature of 25 °C; 70 cycles total). Data were analyzed using Microsoft Excel and GraphPad Prism according to the literature.^107^ DSF assays were performed in independent duplicates, each composed of technical duplicates (Supporting Fig. S4).

### 5.6 Molecular docking studies

Docking studies were performed using four Bcl-2 inhibitors as potential AspH ligands: (*R*)-gossypol (AT-101),^37^, ^38^, ^39^ venetoclax (ABT-199),^43^, ^44^ navitoclax (ABT-263),^49^ and ABT-737.^55^ The structure files of AspH and the four ligands were prepared using the BIOVIA Discovery Studio 2019 (Dassault Systèmes BIOVIA) software suite; the protein structure was based on a deposited AspH crystal structure (PDB ID: 5JZA)^28^ for which all water molecules and the *N-*oxalylglycine (NOG) ligand binding the AspH active site were deleted. AspH residues around the Mn(II) atom (radius = 20 Å) were defined as possible binding sites for ligand docking. Molecular docking studies were then performed by docking the Bcl-2 inhibitors into the defined AspH binding sites using the protein ligand docking software GOLD 5.1 (Cambridge Structural Database)^108^ with a default setting of 100 genetic algorithm (GA) runs for each ligand. For each GA run, a maximum of 125,000 operations was performed; when the top ten solutions possessed root-mean-square deviation (RMSD) values within 1.5 Å, the docking process was terminated. ChemPLP^109^ was used as scoring function. The docking pose for each ligand was analyzed using PyMOL (Supporting Figs. S5–S8).

## Declaration of Competing Interest

The authors declare that they have no known competing financial interests or personal relationships that could have appeared to influence the work reported in this paper.
