# Supplementary material for: Small-molecule active pharmaceutical ingredients of approved cancer therapeutics inhibit human aspartate/asparagine-β-hydroxylase
Source: Bioorg Med Chem. 2020 Oct 15;28(20):115675. doi: 10.1016/j.bmc.2020.115675 (PMC7588595; doi:10.1016/j.bmc.2020.115675)
Supplement: Supplementary data 2 [file mmc2.pdf]

# Supporting Information

## Small-Molecule Active Pharmaceutical Ingredients of Approved Cancer Therapeutics Inhibit Human Aspartate/asparagine-β-hydroxylase

Lennart Brewitz[1], Anthony Tumber[1], Xiaojin Zhang[1,2], and Christopher J. Schofield[1,*]

**Author affiliations**

[1]Chemistry Research Laboratory, University of Oxford, 12 Mansfield Road, OX1 3TA, Oxford, United Kingdom.

[2]Laboratory of Drug Design and Discovery, Department of Chemistry, China Pharmaceutical University, Nanjing 211198, China.

Email: christopher.schofield@chem.ox.ac.uk

**Supporting Figure S1. AspH substrate requirements and structure of the synthetic AspH substrate used in this work.** (a) the non-canonical (Cys 1–2, 3–4, 5–6; left) and the canonical (Cys 1–3, 2–4, 5–6; right) EGFD disulfide patterns, both bearing the consensus sequence (orange/red) for AspH-catalysed Asp/Asn-residue (red) hydroxylation. The non-canonical EGFD disulfide isomer has been identified as a substrate requirement for isolated AspH; the canonical EGFD disulfide isomer was not a substrate;[1] (b) structure of the cyclic thioether peptide hFX-CP$_{101\text{-}119}$[1] which was used in this work as AspH substrate for solid phase extraction (SPE) coupled to mass spectrometry (MS) AspH inhibition assays.[2] The cyclic thioether substrate mimics the non-canonical EGFD disulfide pattern of human coagulation factor X (hFX), which is a reported human AspH substrate;[3] The peptide was synthesized and purified as previously reported.[1,4] The AspH hydroxylation site (Asp103$_{hFX}$) is in red, consensus sequence residues are in orange, the cysteine sulfur is in green, substituted residues are in light blue; numbering is according to the sequence of hFX.

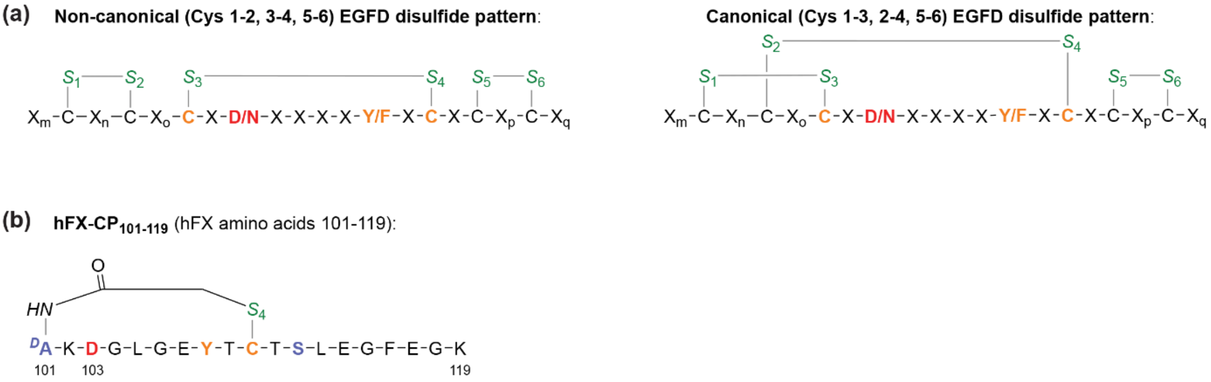

**Supporting Figure S2. AspH inhibition by small-molecule anticancer APIs.** The AspH inhibition assays were performed in independent duplicates as described in the Materials and Methods Section using 50 nM $His_6$-$AspH_{315-758}$, 1 μM hFX-$CP_{101-119}$ (Supporting Figure S1b), 100 μM L-ascorbic acid (LAA), 3 μM 2-oxoglutarate (2OG), and 2 μM ammonium iron(II) sulfate hexahydrate (FAS, $(NH_4)_2Fe(SO_4)_2 \cdot 6H_2O$), in 50 mM HEPES buffer (pH 7.5, 20 °C). The results are shown as the mean average of two independent runs (n = 2; mean ± standard deviation, SD).

Eleven out of 316 compounds of an anticancer active pharmaceutical ingredient (API) compound library were identified to inhibit >80% AspH activity (red line) relative to a negative DMSO control and a positive control using pyridine-2,4-dicarboxylic acid (2,4-PDCA) as the inhibitor. The detailed screening results are shown in the Supporting Data Sheet. The low standard deviation for the inhibition of AspH by most of the compounds among the two independent runs indicates that the majority of the compounds of the anticancer API compound library did not decompose in the DMSO stock solutions. Signal-to-noise (S/N) ratios and Z'-factors were calculated for the two 384-well assay plates according to the literature using Microsoft Excel; Z'-factors >0.5 indicate a stable and robust assay.[5]

Plate 1: S/N = 67.7; Z'-factor: 0.94

Plate 2: S/N = 18.8; Z'-factor: 0.83

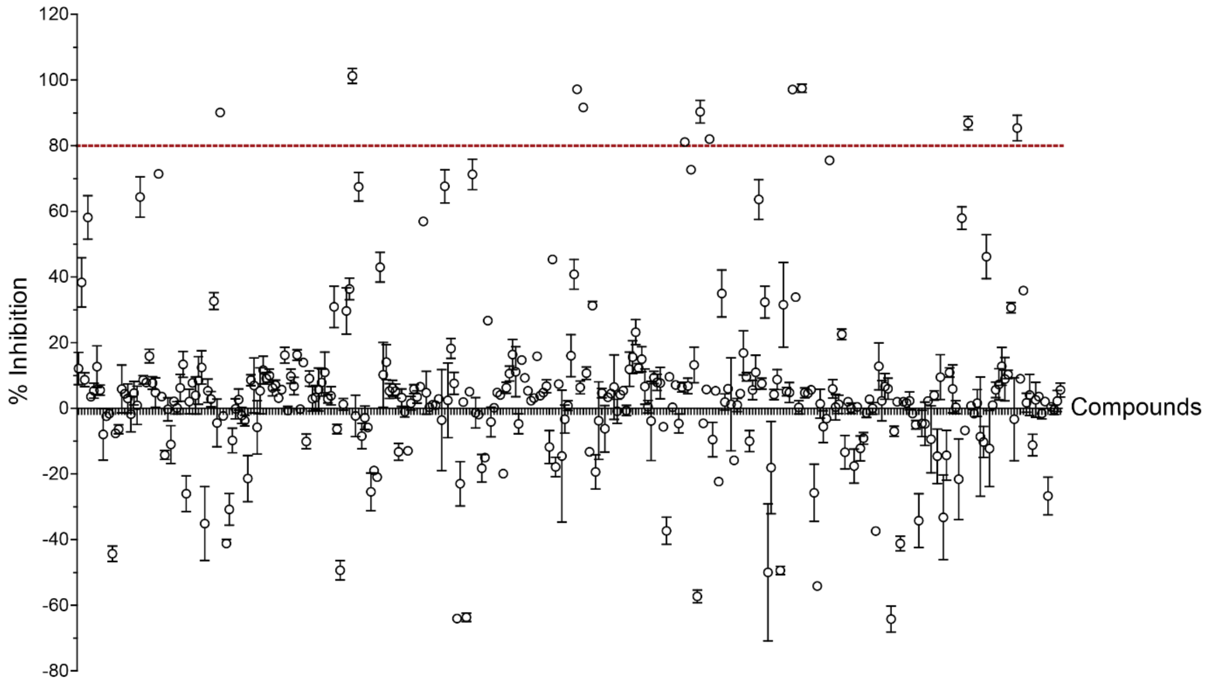

**Supporting Figure S3. Assay robustness.** (a) Z'-factors (circles) and (b) signal-to-noise (S/N) ratios (squares) for the AspH inhibition assay plates analysed in the described work to determine $IC_{50}$-values (16 compounds per plate including DMSO and 2,4-PDCA controls; technical duplicates in were adjacent wells). Z'-factors >0.5 (grey line) indicate a relatively stable and robust assay. S/N ratios and Z'-factors were calculated for the 384-well assay plates according to the literature using Microsoft Excel.[5] Assays were performed as described in the Materials and Methods Section using 50 nM $His_6$-$AspH_{315-758}$ and $hFX\text{-}CP_{101-119}$ as an AspH substrate (Supporting Figure S1b).

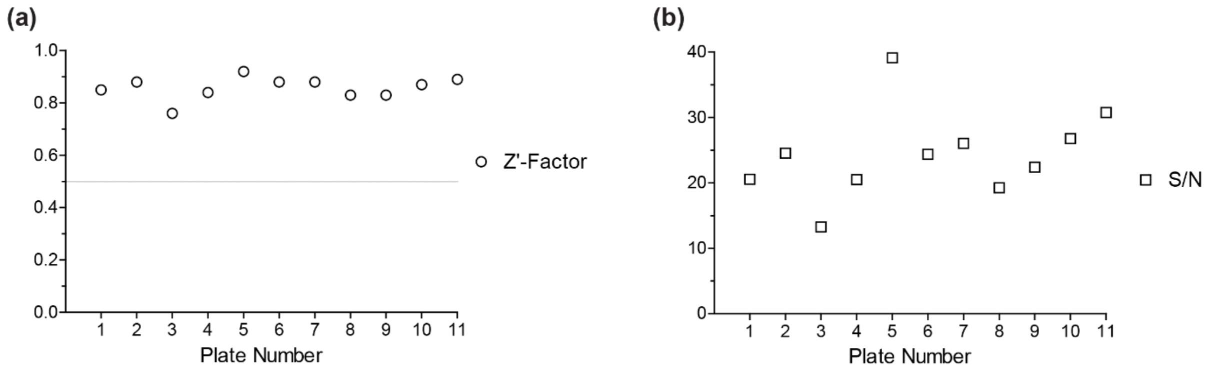

**Supporting Figure S4. Effect of small-molecule AspH inhibitors on the AspH melting temperature.** Shifts ($\Delta T_m$) of the AspH melting temperature ($T_m$) were assayed using differential scanning fluorimetry (DSF) assays as described in the Materials and Methods Section and are given with respect to DMSO controls. The validated AspH inhibitor 2,4-PDCA, of which crystal structures in complex with AspH have been reported,[2] shows a notable increase in the AspH $T_m$ ($\Delta T_m$ ~ 3.2°C; Entry 1), in agreement with a prior report ($\Delta T_m$ = 3.5 ± 0.6°C).[2] With the possible exception of navitoclax (ABT-263),[6] the results do not provide evidence that the identified AspH inhibitors are strong binders of AspH. Navitoclax apparently destabilizes AspH ($\Delta T_m$ ~ -2.1°C; Entry 8), while the other Abbott-developed Bcl-2 inhibitors venetoclax[7-8] (ABT-199; $\Delta T_m$ ~ 0.7°C; Entry 4) and ABT-737[9] ($\Delta T_m$ ~ 1.5°C; Entry 12) apparently stabilize AspH as higher AspH $T_m$ indicate. Conclusive $\Delta T_m$ data for the AspH inhibitors (*R*)-gossypol (AT-101), midostaurin (PKC412), tubacin, obatoclax (GX15-070), and vemurafenib (PLX4032) could not be obtained, due to broad melting curves or due to intrinsic compound fluorescence interfering with the excitation/emission wavelengths of SYPRO orange.

| **Entry** | **AspH-Inhibitor** | [a] **$T_m$-shift** [°C] |
|---|---|---|
| 1 | 2,4-PDCA | 3.2 ± 0.4 |
| 2 | (*R*)-Gossypol (AT-101) | [b] - |
| 3 | Bleomycin $A_2$ | -0.8 ± 0.4 |
| 4 | Venetoclax (ABT-199) | 0.7 ± 0.3 |
| 5 | Belinostat (PXD101) | 0.1 ± 0.2 |
| 6 | Midostaurin (PKC412) | [b] - |
| 7 | Tubacin | [b] - |
| 8 | Navitoclax (ABT-263) | -2.1 ± 0.3 |
| 9 | Mithramycin A (plicamycin) | 0.4 ± 0.2 |
| 10 | Obatoclax (GX15-070) | [b] - |
| 11 | Vemurafenib (PLX4032) | [b] - |
| 12 | ABT-737 | 1.5 ± 0.1 |

a) Mean average of two independent runs each composed of technical duplicates (n = 2; mean ± standard deviation, SD); b) data not analysed because of broad melting curves or because of the interference of the intrinsic compound fluorescence with the excitation/emission wavelengths of SYPRO orange.

**Supporting Figure S5. Docking study on the potential binding mode of (*R*)-gossypol (AT-101) with the AspH substrate binding pocket.** Colors: violet/gray: His$_6$-AspH$_{315-758}$; yellow: carbon-backbone of (*R*)-gossypol; light green: carbon-backbone of the non-canonical (Cys 1–2, 3–4, 5–6) disulfide isomer of the hFX-EGFD1$_{86-124}$ peptide;[1] red: oxygen; blue: nitrogen; dark yellow: sulfur. Molecular docking studies were performed using GOLD 5.1[10] and the AspH crystal structure (PDB ID: 5JZA)[1] as described in the Materials and Methods Section.

(a) AspH surface representation for docked (*R*)-gossypol. The comparison to (b) substrate (hFX-EGFD1$_{86-124}$) binding as observed in the AspH:hFX-EGFD1$_{86-124}$ (PDB ID: 5JZ8)[1] structure suggests that (*R*)-gossypol could bind in the substrate binding pocket. Close-up views (c and d) indicate that (*R*)-gossypol might bind in a manner positioned to π-π-stack with Phe529 and to interact with His530 (3.1 Å). The hFX-EGFD1$_{86-124}$ substrate peptide interacts with different residues of the AspH oxygenase and TPR domains.

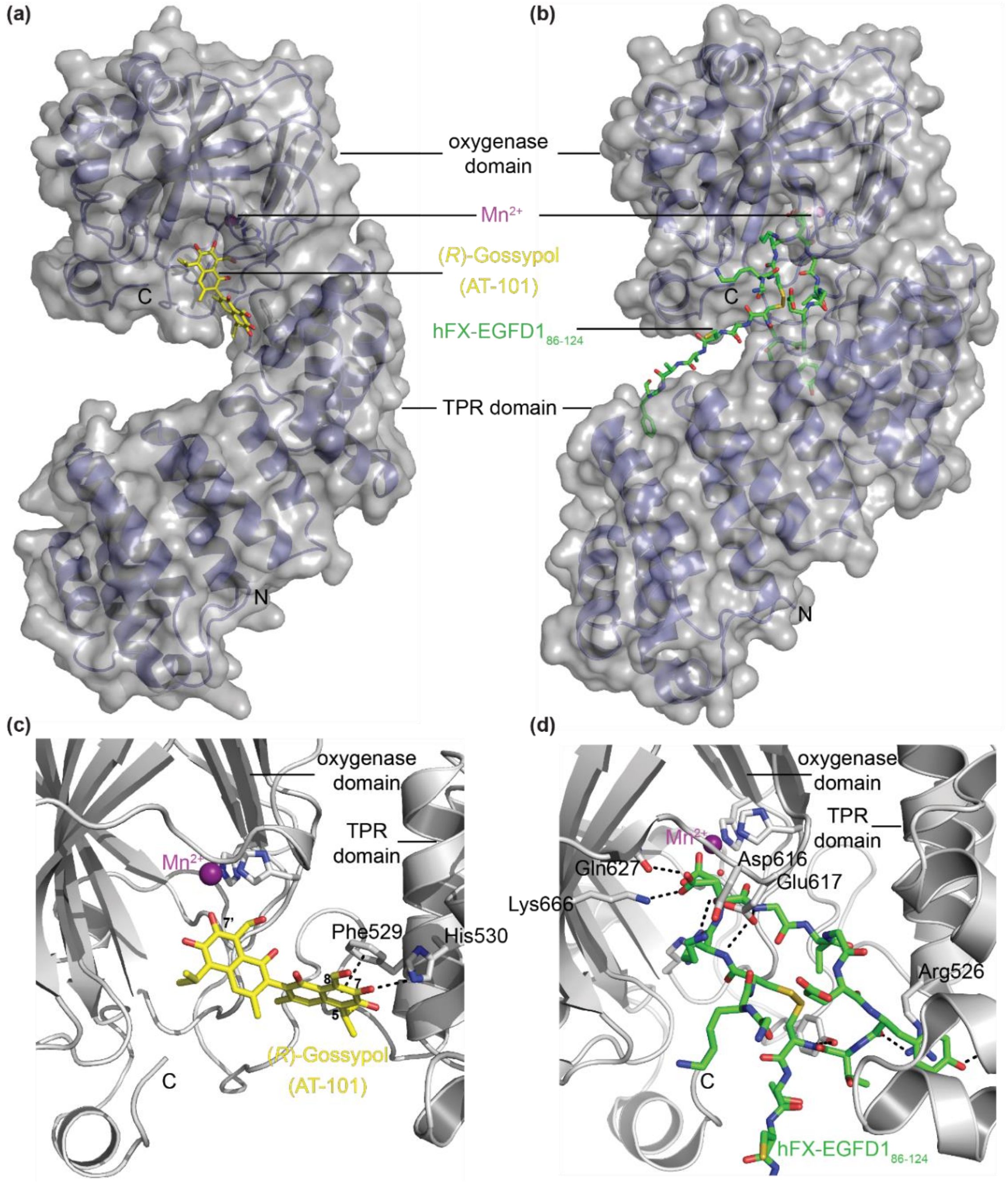

**Supporting Figure S6. Docking studies on the potential binding modes of reported small-molecule Bcl-2 inhibitors with the AspH substrate binding pocket.** Colors: violet/gray: His$_6$-AspH$_{315\text{-}758}$; magenta: carbon-backbone of (a) venetoclax[7-8] (ABT-199); orange: carbon-backbone of (b) navitoclax[6] (ABT-263); maroon: carbon-backbone of (c) ABT-737;[9] light green: carbon-backbone of the non-canonical (Cys 1–2, 3–4, 5–6) disulfide isomer of the hFX-EGFD1$_{86\text{-}124}$ peptide;[1] purple: Mn; red: oxygen; blue: nitrogen; yellow: sulfur; green: chlorine; light blue: fluorine. Molecular docking studies were performed using GOLD 5.1[10] and the AspH crystal structure (PDB ID: 5JZA)[1] as described in the Materials and Methods Section.

AspH surface representations for the three docked Abbott-developed Bcl-2 inhibitors (a) venetoclax, (b) navitoclax, and (c) ABT-737. Their comparison to (d) substrate (hFX-EGFD1$_{86\text{-}124}$) binding as observed in the AspH:hFX-EGFD1$_{86\text{-}124}$ (PDB ID: 5JZ8)[1] structure suggests that the three inhibitors could bind in the substrate binding pocket.

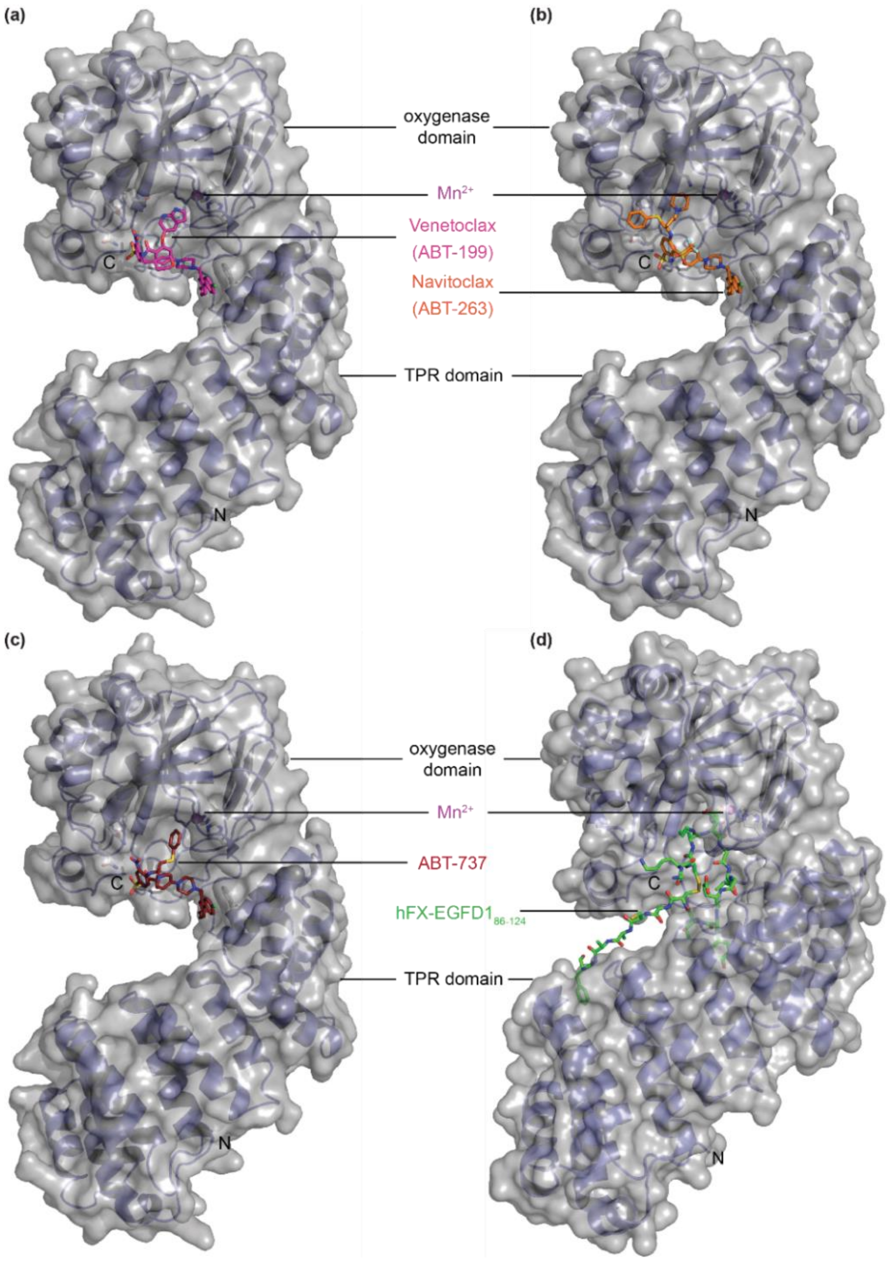

**Supporting Figure S7. Docking studies suggest that three reported small-molecule Bcl-2 inhibitors may bind to the AspH substrate binding pocket.** Colors: gray: $His_6$-$AspH_{315-758}$; magenta: carbon-backbone of (a) venetoclax[7-8] (ABT-199); orange: carbon-backbone of (b) navitoclax[6] (ABT-263); maroon: carbon-backbone of (c) ABT-737;[9] light green: carbon-backbone of the non-canonical (Cys 1–2, 3–4, 5–6) hFX-$EGFD1_{86-124}$ peptide;[1] purple: Mn; red: oxygen; blue: nitrogen; yellow: sulfur; green: chlorine; light blue: fluorine. Molecular docking studies were performed using GOLD 5.1[10] and the AspH crystal structure (PDB ID: 5JZA)[1] as described in the Materials and Methods Section.

Close-up views of three Abbott-developed Bcl-2 inhibitors (a) venetoclax, (b) navitoclax, and (c) ABT-737 docked into AspH. Their comparison to (d) substrate (hFX-$EGFD1_{86-124}$) binding as observed in the AspH:hFX-$EGFD1_{86-124}$ (PDB ID: 5JZ8)[1] structure suggests that the three inhibitors could interact with AspH residues positioned close to the AspH substrate binding pocket including the TPR domain. The docking studies suggest that these inhibitors do not interact with residues of the AspH acidic loop (Asp616, Glu617) which are positioned to interact with the hFX-$EGFD1_{86-124}$-substrate as evidenced by the AspH:hFX-$EGFD1_{86-124}$ crystal structure.[1]

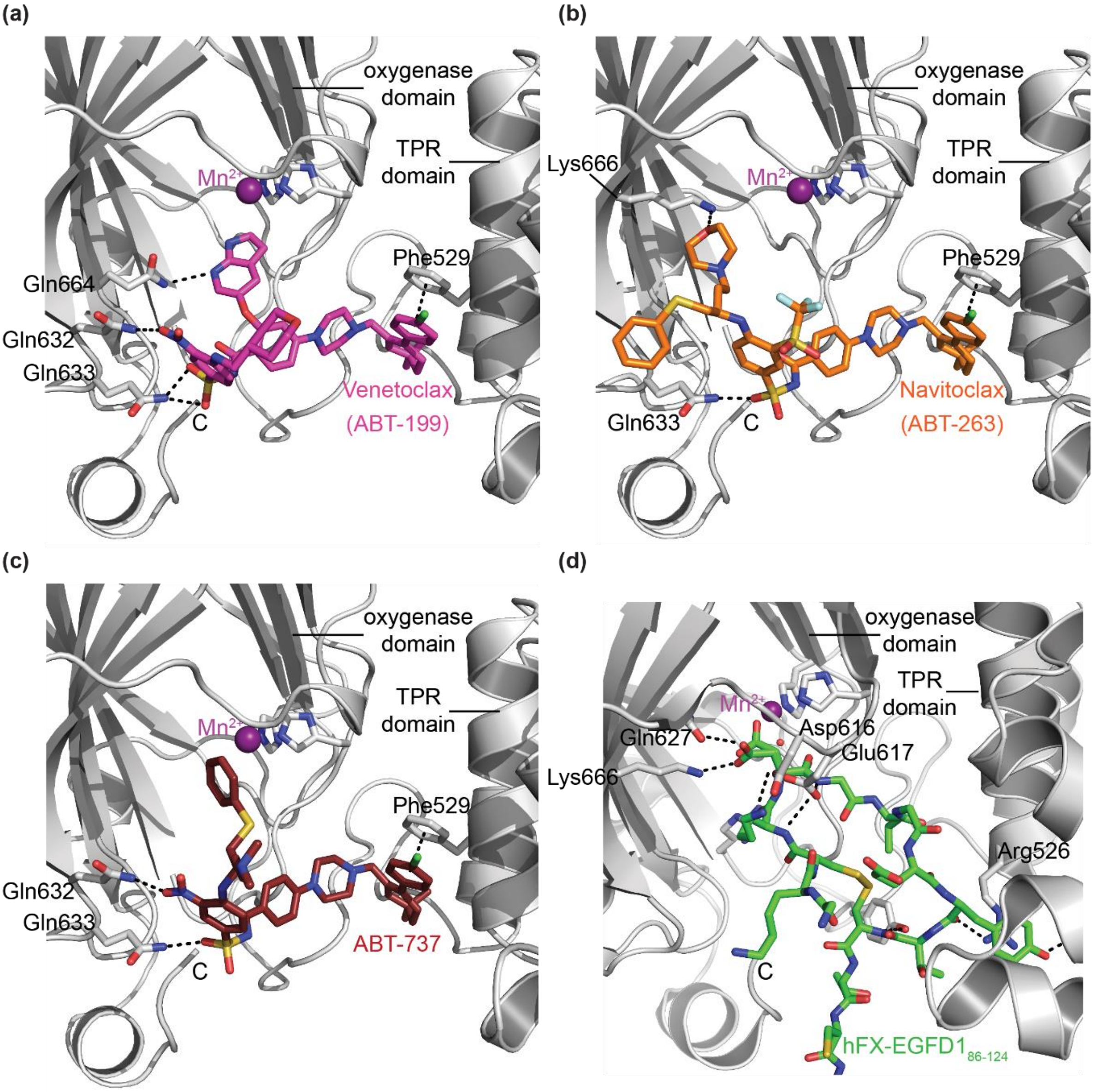

**Supporting Figure S8. Comparison of the predicted AspH binding modes of four different Bcl-2 inhibitors.** Colors: gray: $His_6$-$AspH_{315\text{-}758}$; yellow: carbon-backbone of (*R*)-gossypol (a); magenta: carbon-backbone of venetoclax (b); orange: carbon-backbone of navitoclax (c); maroon: carbon-backbone of ABT-737 (d); red: oxygen; blue: nitrogen; dark yellow: sulfur; green: chlorine; light blue: fluorine. Molecular docking studies were performed using GOLD 5.1[10] and the AspH crystal structure (PDB ID: 5JZA)[1] as described in the Materials and Methods Section and in Supporting Figures S5-7.

The docking studies suggest that the Bcl-2 inhibitors might interact with AspH through a π-π-stacking interaction with Phe529 (3.9 Å). The hydroxyl group at the C-7 position of (*R*)-gossypol is predicted to interact with His530 (3.1 Å) and the iso-propyl substituent at the C-5 position of (*R*)-gossypol is predicted to bind to a hydrophobic pocket formed by Leu564, Phe556, and Phe529. The C-7' hydroxyl group of (*R*)-gossypol is within 4.2 Å distance of the AspH active site metal. An induced fit mechanism was previously observed for AspH upon substrate binding (Supporting Figures S5 and S6),[1] which, if occurring upon (*R*)-gossypol binding to AspH, might result in a further decrease of the metal-inhibitor distance. The Bcl-2 inhibitors venetoclax, navitoclax, and ABT-737 are predicted to interact with Gln633 (venetoclax: 2.6/2.8 Å; navitoclax: 2.5 Å; ABT-737: 2.7 Å). Venetoclax and ABT-737 are also predicted to interact with Gln632 (2.7 and 2.6 Å, respectively), venetoclax with Gln664 (3.2 Å), and navitoclax through its morpholine oxygen atom with Lys666 (2.7 Å), which was previously shown to engage in EGFD substrate binding (Supporting Figure S5d).[1] The predicted interactions of ABT-737 with AspH resemble those of venetoclax rather than navitoclax, potentially rationalizing the observed different efficiencies by which venetoclax and ABT-737 inhibit AspH (venetoclax is about three times more efficient than ABT-737; Table 1): ABT-737 might interact with AspH through only one sulfone oxygen atom and might not interact with Gln664. The PLP fitness scores were: (a) 70.1; (b) 87.7; (c) 97.7; (d) 89.1. The PLP fitness score for navitoclax was the highest among all four Bcl-2 inhibitors (97.7), indicative of the best docking fit.

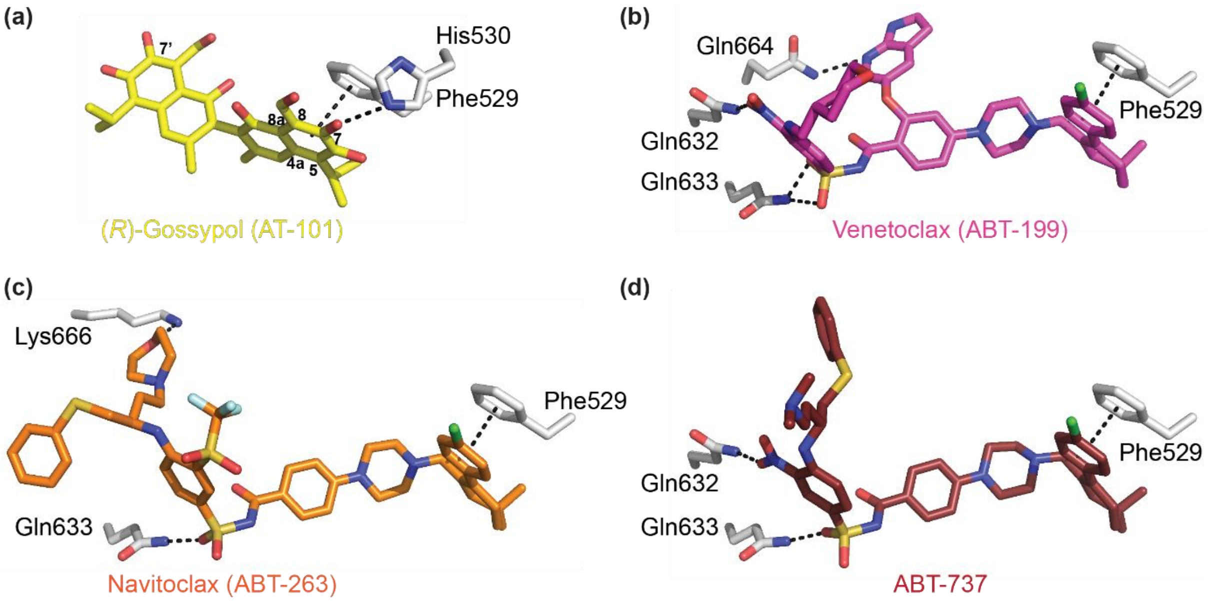

**Supporting References**


1. Pfeffer I, Brewitz L, Krojer T, Jensen SA, Kochan GT, Kershaw NJ, Hewitson KS, McNeill LA, Kramer H, Münzel M, Hopkinson RJ, Oppermann U, Handford PA, McDonough MA, Schofield CJ. Aspartate/asparagine-β-hydroxylase crystal structures reveal an unexpected epidermal growth factor-like domain substrate disulfide pattern. *Nat Commun.* 2019;10:4910.
2. Brewitz L, Tumber A, Pfeffer I, McDonough MA, Schofield CJ. Aspartate/asparagine-β-hydroxylase: a high-throughput mass spectrometric assay for discovery of small molecule inhibitors. *Sci Rep.* 2020;10:8650.
3. McMullen BA, Fujikawa K, Kisiel W, Sasagawa T, Howald WN, Kwa EY, Weinstein B. Complete amino acid sequence of the light chain of human blood coagulation factor X: evidence for identification of residue 63 as β-hydroxyaspartic acid. *Biochemistry.* 1983;22:2875-2884.
4. Brewitz L, Tumber A, Schofield CJ. Kinetic parameters of human aspartate/asparagine-β-hydroxylase suggest that it has a possible function in oxygen sensing. *J Biol Chem.* 2020;295:7826-7838.
5. Zhang J-H, Chung TDY, Oldenburg KR. A simple statistical parameter for use in evaluation and validation of high throughput screening assays. *J Biomol Screen.* 1999;4:67-73.
6. Tse C, Shoemaker AR, Adickes J, Anderson MG, Chen J, Jin S, Johnson EF, Marsh KC, Mitten MJ, Nimmer P, Roberts L, Tahir SK, Xiao Y, Yang X, Zhang H, Fesik S, Rosenberg SH, Elmore SW. ABT-263: a potent and orally bioavailable Bcl-2 family inhibitor. *Cancer Res.* 2008;68:3421-3428.
7. Souers AJ, Leverson JD, Boghaert ER, Ackler SL, Catron ND, Chen J, Dayton BD, Ding H, Enschede SH, Fairbrother WJ, Huang DCS, Hymowitz SG, Jin S, Khaw SL, Kovar PJ, Lam LT, Lee J, Maecker HL, Marsh KC, Mason KD, Mitten MJ, Nimmer PM, Oleksijew A, Park CH, Park C-M, Phillips DC, Roberts AW, Sampath D, Seymour JF, Smith ML, Sullivan GM, Tahir SK, Tse C, Wendt MD, Xiao Y, Xue JC, Zhang H, Humerickhouse RA, Rosenberg SH, Elmore SW. ABT-199, a potent and selective BCL-2 inhibitor, achieves antitumor activity while sparing platelets. *Nat Med.* 2013;19:202-208.
8. Ku Y-Y, Chan VS, Christesen A, Grieme T, Mulhern M, Pu Y-M, Wendt MD. Development of a convergent large-scale synthesis for venetoclax, a first-in-class BCL-2 selective inhibitor. *J Org Chem.* 2019;84:4814-4829.
9. Oltersdorf T, Elmore SW, Shoemaker AR, Armstrong RC, Augeri DJ, Belli BA, Bruncko M, Deckwerth TL, Dinges J, Hajduk PJ, Joseph MK, Kitada S, Korsmeyer SJ, Kunzer AR, Letai A, Li C, Mitten MJ, Nettesheim DG, Ng S, Nimmer PM, O'Connor JM, Oleksijew A, Petros AM, Reed JC, Shen W, Tahir SK, Thompson CB, Tomaselli KJ, Wang B, Wendt MD, Zhang H, Fesik SW, Rosenberg SH. An inhibitor of Bcl-2 family proteins induces regression of solid tumours. *Nature.* 2005;435:677-681.
10. Jones G, Willett P, Glen RC, Leach AR, Taylor R. Development and validation of a genetic algorithm for flexible docking. *J Mol Biol.* 1997;267:727-748.
